# The pan-immune-inflammation value predicts stroke-associated pneumonia and poor outcome in spontaneous intracerebral hemorrhage: a machine learning approach

**DOI:** 10.3389/fneur.2026.1906754

**Published:** 2026-09-17

**Authors:** Anni Chen, Jiahui Wang, Qiaoying Huang, Chaoxiong Shen, Zhizhou Hu, Xiaohong Hu

**Affiliations:** Department of Neurology, Longyan First Affiliated Hospital of Fujian Medical University, Longyan, Fujian, China

**Keywords:** cerebral hemorrhage, machine learning, pan-immune-inflammation value, stroke, stroke-associated pneumonia

## Abstract

**Objective:**

Stroke-associated pneumonia (SAP) constitutes a major complication following spontaneous intracerebral hemorrhage (sICH), posing a significant clinical challenge for accurate prediction. This study aimed to evaluate whether integrating the pan-immune-inflammation value (PIV) enhances the predictive performance of machine learning (ML) models for SAP and poor functional outcome.

**Methods:**

A retrospective cohort of 371 sICH patients was analyzed. Feature selection was performed using the least absolute shrinkage and selection operator (LASSO). Predictive models for SAP and poor outcome [modified Rankin Scale score (mRS) > 2 at 90 days] were developed and compared across nine ML algorithms. Model performance was assessed by discrimination (area under the receiver operating characteristic curve, AUC), calibration, and decision curve analysis (DCA). Interpretability was achieved via SHapley Additive exPlanations (SHAP).

**Results:**

Elevated PIV was independently associated with SAP [odds ratio (OR) 13.55, 95% confidence interval (CI) 4.14–44.39; *p* < 0.0001] and poor 90-day functional outcome (OR 25.27, 95% CI 7.75–82.34; *p* < 0.0001). Among the algorithms, extreme Gradient Boosting (XGBoost) and logistic regression (LR) demonstrated the highest predictive performance for poor outcome (AUC 0.912) and SAP (AUC 0.856), respectively. Incorporating PIV significantly improved discriminatory ability (XGBoost: 0.926–0.942; LR: 0.905–0.931; both *p* < 0.05). The models demonstrated good calibration, provided net clinical benefit on DCA, and were interpretable through SHAP analysis, which consistently identified PIV as a critical predictor.

**Interpretation:**

The integration of PIV into interpretable ML models significantly improves the accuracy of predicting SAP and functional outcome after sICH. This strategy, combining a systemic inflammatory biomarker with explainable ML, holds promise for advancing personalized risk stratification in neurocritical care.

## Introduction

SAP defined as pneumonia occurring within the first week after stroke, is a major complication that poor functional outcome (1). This risk is especially pronounced following sICH—the most lethal stroke subtype (2). Patients with sICH exhibit a notably high incidence of SAP, which is strongly associated with increased mortality and morbidity (3–6).

According to the data from the 2019 Global Burden of Disease (GBD 2019) (2), sICH accounts for 27.9% of all stroke cases and is the most lethal among all stroke subtypes. SAP occurs in approximately 14–27.8% of patients, and the incidence of pneumonia is relatively higher in patients with sICH than in those with ischemic stroke (3).

However, current prophylactic strategies, primarily antibiotic-based, have shown limited efficacy in trials (7, 8), underscoring the need for better risk stratification tools grounded in a deeper understanding of the underlying pathophysiology. A key mechanism in this context is stroke-induced immtmodepression syndrome (SIDS), an acute-phase state of systemic immune dysregulation that critically weakens host defenses and predisposes patients to infections such as SAP (9, 10). Therefore, identifying a biomarker that can accurately and comprehensively reflect this systemic immune-inflammatory state is crucial for enabling early, targeted interventions.

PIV integrating neutrophil, platelet, monocyte, and lymphocyte counts, has emerged as a robust, holistic indicator of immune-inflammatory networks, surpassing conventional ratios in prognostic utility across various diseases (11–13). However, its predictive value for SAP after sICH remains unknown. To rigorously evaluate such a complex biomarker, advanced analytical methods are required. ML algorithms are ideally suited for this task due to their capacity to model non-linear relationships and complex interactions within high-dimensional clinical data, potentially offering superior predictive performance over traditional statistical models (14–18).

Therefore, this study hypothesizes that integrating PIV into ML models will significantly enhance the prediction of SAP and poor functional outcome post-ICH. To ensure a comprehensive and robust evaluation within our development cohort, we employed nine distinct ML algorithms—including XGBoost, LR, Support Vector Machine (SVM), K-Nearest Neighbors (KNN), Naive Bayes (NB), Decision Tree (DT), Random Forest (RF), Adaptive Boosting (AdaBoost), and Gradient Boosting Machine (GBM). This selection was designed to cover a wide spectrum of methodological approaches, encompassing both linear and non-linear modeling paradigms, allowing for a comparative evaluation of their relative performance and internal consistency across different algorithmic families within this single-center cohort. Through this comparative framework, we aimed to evaluate the added value of PIV and identify the optimal predictive model as a foundation for future external validation and potential clinical translation.

## Methods

The present clinical study was conducted in accordance with the Declaration of Helsinki and was approved by the Scientific Research Ethics Committee of Longyan First Hospital (approval no. LYREC2025-K158-01). All participants provided written informed consent to participate.

### Study population

This study retrospectively collected data from 371 patients diagnosed with sICH at Longyan First Hospital between December 2022 and May 2025. The inclusion criteria were: (1) age 18 or older; (2) diagnosed with spontaneous sICH through CT scans; (3) admitted within 24 h of symptom onset. The exclusion criteria included: (1) diagnosed with pneumonia, active infection, or fever within 2 weeks prior to admission; (2) secondary sICH due to brain injury, aneurysm, tumor, or arteriovenous malformation; (3) hemorrhagic transformation after ischemic stroke; (4) history of malignant tumors, hematological diseases, or severe liver and kidney conditions; (5) use of immunosuppressants for more than 3 months; (6) invasive procedures such as neurosurgical intervention or mechanical ventilation before the diagnosis of SAP.

### Data collection

(1) Demographic data (sex and age); (2) medical history [hypertension, diabetes, acute coronary syndrome (ACS), chronic obstructive pulmonary disease (COPD), atrial fibrillation, hyperlipidemia], medication history, smoking and alcohol consumption history; (3) clinical data [baseline Glasgow Coma Scale (GCS), National Institute of Health stroke scale (NIHSS), Intracerebral hemorrhage-associated pneumonia score A (ICH-APS-A score) (19), enteral nutrition (EN), dysphagia, mRS score at day 90, length of hospital stay]; (4) imaging characteristics [baseline hematoma volume (infratentorial hemorrhage volume ≥10 mL or Supratentorial hemorrhage volume ≥40 mL) (19), hematoma location (Supratentorial hemorrhage), and intraventricular hemorrhage]; (5) laboratory test variables [neutrophil count (NEU), lymphocyte count (LYM), monocyte count (MONO), platelet count (PLT), total protein (TP), albumin (ALB), alanine transaminase (ALT), aspartate transaminase (AST), total bilirubin (TBIL), indirect bilirubin (IBIL), APOA, APOB, creatinine (CR), blood urea nitrogen (BUN), uric acid (UA), blood glucose (GLU), Na+, K+, Cl−, Mg2+, Ca2+, total cholesterol (TC), triglycerides (TG), high-density lipoprotein (HDL), low-density lipoprotein (LDL), red blood cell count (RBC), hemoglobin (Hb), white blood cell count (WBC), eosinophil count (EOS), INR, fibrinogen (Fib)]; (6) composite inflammation index (PIV, pan-immune inflammation value; NLR, neutrophil-to-lymphocyte ratio; MLR, mononuclear-to-lymphocyte ratio; PLR, platelet-to-lymphocyte ratio). PIV = (monocytes × neutrophils × platelets) ÷ lymphocytes.

### Clinical outcomes

The primary endpoints were the occurrence of SAP and poor functional outcome at 90 days (mRS > 2). SAP was defined as a lower respiratory tract infection occurring within the first 7 days after stroke onset, in accordance with the 2015 consensus criteria (1). The diagnosis required the presence of new pulmonary infiltrates on chest imaging in conjunction with compatible clinical features (such as fever, purulent sputum, or leukocytosis), supported by documented antibiotic treatment. Functional outcome was assessed using the mRS at 90 days post-sICH, with scores of 0–2 categorized as a good outcome and scores of 3–6 as a poor outcome.

### Statistical analysis

A retrospective analysis was conducted on 371 patients with sICH. Participants were stratified into two cohorts based on the diagnosis of SAP. Continuous variables are presented as mean ± standard deviation or median with interquartile range, depending on data distribution. Categorical variables are expressed as frequencies and percentages. To enhance clinical applicability, age was dichotomized using a threshold of 65 years, informed by global burden of disease data indicating elevated risk in this population (20, 21). Inter-group comparisons for categorical and continuous variables were performed using the Chi-square test and the Student’s *t*-test or Mann–Whitney *U* test, respectively. The PIV was analyzed by dividing participants into quintiles (Q1–Q5). A multivariate binary logistic regression model was then employed to identify independent factors associated with SAP and poor functional outcome (mRS > 2), with PIV quintiles as the primary exposure variable. The internal consistency of these findings within this cohort was further examined through subgroup and interaction analyses.

Predictor variables for model construction were selected using LASSO regression (22, 23). Nine machine learning algorithms—XGBoost, LR, SVM, KNN, NB, DT, RF, AdaBoost, and GBM—were trained and compared to ensure a comprehensive evaluation across different modeling approaches. To prevent overfitting and optimize generalizability, a rigorous 10-fold cross-validation framework was implemented for model training. Model performance was evaluated primarily by AUC, supplemented by sensitivity, specificity, accuracy, and F1-score. To ensure a comprehensive and unbiased evaluation, nine ML algorithms were selected to represent diverse modeling paradigms: linear (LR), tree-based ensemble (XGBoost, RF, AdaBoost, GBM), kernel-based (SVM), instance-based (KNN), probabilistic (NB), and rule-based (DT). This diversity was intended to avoid over-reliance on any single methodological family and to balance predictive power with interpretability. The final model for each outcome was not prespecified but was empirically determined by comparing AUC across all algorithms. All models were trained using 10-fold cross-validation, with hyperparameter tuning performed within each fold to prevent overfitting. LASSO regression was used for variable selection, and model performance was further evaluated by calibration and DCA to ensure clinical utility. The optimal-performing algorithm for each clinical outcome was selected as the baseline model. The variable PIV was then integrated to build the final predictive model. The model’s calibration was assessed using the Hosmer–Lemeshow test and calibration curves, while its clinical utility was quantified via DCA. Finally, to ensure interpretability, SHAP were applied to elucidate the contribution of key predictors, including PIV, to the model’s outputs (24–26).

All statistical analyses were performed using R software (version 4.2.2) and SPSS (version 22.0), with a two-tailed *p*-value of <0.05 considered statistically significant.

## Results

This study included a total of 371 participants. Supplementary Table 1 shows that 125 (33.69%) of the sICH patients developed SAP, with 227 (61.19%) being male. In the SAP group, 85 (68.00%) were aged 65 or older, compared to 102 (41.46%) in the non-SAP group (*p* < 0.0001). The SAP group had a higher proportion of smokers (39.20% vs. 27.64%, *p* = 0.03) and drinkers (47.20% vs. 28.46%, *p* < 0.001). Compared to the non-SAP group, the SAP group had a higher prevalence of COPD (9.60% vs. 0.81%, *p* < 0.0001); more SAP patients experienced dysphagia (27.20% vs. 10.16%, *p* < 0.0001) and required enteral nutrition (45.60% vs. 15.85%, *p* < 0.0001). At admission, the SAP group had a higher proportion of patients with low GCS scores and high NIHSS scores, as well as a higher ICH-APS-A score (GCS score: 12.00% vs. 1.63%; NIHSS score: 37.60% vs. 9.76%; ICH-APS-A score: 7.81 ± 3.28 vs. 3.66 ± 2.41, *p* < 0.0001). The proportion of patients with a high mRS score at discharge was higher in the SAP group compared to the non-SAP group (64% vs. 18.29%, *p* < 0.0001). In terms of imaging characteristics, SAP patients had a larger baseline hematoma volume (36.8% vs. 2.43%, *p* < 0.01) and a higher rate of IVH (54.40% vs. 30.08%, *p* < 0.0001). The SAP group had higher baseline inflammation composite indices (PIV, NLR, MLR, PLR) and other laboratory indicators (WBC, NEU, LYM, MONO, PLT) (*p* < 0.05) with lower levels of baseline Mg2+, APOB, EOS (*p* < 0.05). Compared to the non-SAP group, the SAP group had a longer hospital stay (24.03 ± 23.97 vs. 17.42 ± 16.25, *p* < 0.01).

This investigation categorized PIV into five distinct groups (Q1-Q5) and subsequently performed a multivariate binary logistic regression analysis on the selected research variables. Table 1 presents the adjusted outcomes of a multivariate binary logistic regression model, which examines the occurrence of PIV and SAP. Model 1 established SAP occurrence as the dependent variable, with adjustments for age and sex. Model 2 built upon Model 1 by further incorporating adjustments for hypertension, diabetes, EN, dysphagia, atrial fibrillation, COPD, history of smoking and drinking. Model 3, extending from Model 2, included additional adjustments for baseline GCS score, NIHSS score, ICH-APS-A score, mRS score at day 90, hematoma location, intraventricular hemorrhage, volume of hematoma, APOB, Mg2+, white blood cell count. Table 2 presents the adjusted outcomes of a multivariate binary logistic regression model, which examines the occurrence of PIV and the incidence of poor functional outcome. The analysis for poor functional outcome followed a similar pattern, with Model 1 adjusting for age and sex; Model 2 further incorporating adjustments for hypertension, diabetes, hyperlipidemia, atrial fibrillation, COPD, history of smoking and drinking, and the utilization of antiplatelet and anticoagulant medications; Model 3 including additional adjustments for baseline GCS score, NIHSS score, occurrence of SAP, hematoma location, intraventricular hemorrhage, volume of hematoma, albumin levels, blood glucose levels and hemoglobin levels. The findings indicated that, irrespective of adjustments, elevated PIV was significantly correlated with SAP (OR 13.55, 95% CI 4.14–44.39; *p* < 0.0001) and poor functional outcome (OR 25.27, 95% CI 7.75–82.34; *p* < 0.0001) in patients with spontaneous intracerebral hemorrhage.

**Table 1:** The binary logistic regression model was constructed based on the occurrence of SAP.

SAP	PIVQ							
	Crude model		Model 1		Model 2		Model 3	
Character	95%CI	*p*	95%CI	*p*	95%CI	*p*	95%CI	*p*
Q2	Ref		Ref		Ref		Ref	
Q1	1.56 (0.62, 3.92)	0.34	1.47 (0.57, 3.77)	0.42	1.48(0.50, 4.34)	0.48	1.32 (0.42, 4.15)	0.64
Q3	1.56 (0.62, 3.92)	0.34	1.57 (0.61, 4.04)	0.35	1.62 (0.52, 4.99)	0.40	0.91 (0.25, 3.31)	0.89
Q4	10.17 (4.41, 23.46)	**<0.0001**	11.07 (4.63, 26.48)	**<0.0001**	21.51 (7.62, 60.68)	**<0.0001**	7.05 (1.98, 25.09)	**0.003**
Q5	12.77 (5.50, 29.63)	**<0.0001**	13.44 (5.61, 32.21)	**<0.0001**	24.42 (8.79, 67.88)	**<0.0001**	14.28 (4.56, 44.71)	**<0.0001**
*p* for trend		**<0.0001**		**<0.0001**		**<0.0001**		**<0.0001**

Crude model: PIVQ; model 1: PIVQ, age, sex; model 2: PIVQ, age, sex, hypertension, fibration, EN, dysphagia, diabetes, COPD, drink, smoke; model 3: PIVQ, age, sex, hypertension, fibration, EN, dysphagia, diabetes, COPD, drink, smoke, GCS, NIHSS, APS, WBC, Mg2+, APOB, location, IVH, volume. PIVQ, PIV quintiles (Q1–Q5); *p* for trend, it represents the *p* value for linear trend across PIV quintiles (Q1–Q5). Bold values indicate statistical significance (*P* < 0.05).

**Table 2:** The binary logistic regression model was constructed based on the poor functional outcome.

mRS	PIVQ							
	Crude model		Model 1		Model 2		Model 3	
Character	95%CI	*p*	95%CI	*p*	95%CI	*p*	95%CI	*p*
Q2	Ref		Ref		Ref		Ref	
Q1	0.18 (0.04, 0.85)	**0.03**	0.18 (0.04, 0.83)	**0.03**	0.1 (0.02, 0.55)	**0.01**	0.11 (0.02, 0.64)	**0.01**
Q3	1.26 (0.51, 3.12)	0.62	1.24 (0.50, 3.09)	0.64	1.27 (0.47, 3.45)	0.64	1.02 (0.32, 3.26)	0.97
Q4	7.65 (3.41, 17.15)	**<0.0001**	7.32 (3.24, 16.55)	**<0.0001**	8.38(3.47, 20.22)	**<0.0001**	5.04 (1.82, 13.99)	**0.002**
Q5	30.5(12.46, 74.67)	**<0.0001**	30.81(12.45, 76.21)	**<0.0001**	43.14(16.15, 115.26)	**<0.0001**	25.27 (7.75, 82.34)	**<0.0001**
*p* for trend		**<0.0001**		**<0.0001**		**<0.0001**		**<0.0001**

Crude model: PIVQ; model 1: PIVQ, age, sex; model 2: PIVQ, age, sex, hypertension, fibration, hyperlipemia, diabetes, COPD, drink, smoke, Antiplate, Anticog; model 3: PIVQ, age, sex, hypertension, fibration, hyperlipemia, diabetes, COPD, drink, smoke, Antiplate, Anticog, GCS, NIHSS, SAP, ALB, GLU, Hb, location, IVH, volume. PIVQ, PIV quintiles (Q1–Q5); *p* for trend, it represents the *p* value for linear trend across PIV quintiles (Q1–Q5). Bold values indicate statistical significance (*P* < 0.05).

As depicted in Figure 1, the correlation between PIV and the occurrence of SAP, along with poor functional outcome in patients with spontaneous intracerebral hemorrhage, were examined using RCS. The findings indicated that PIV and the occurrence of SAP, as well as poor functional outcome in these patients, exhibited a curvilinear relationship. Furthermore, the incidence of SAP and poor functional outcome increased in a nonlinear fashion with the rise in PIV (*p*-nonlinear <0.001).

**Figure 1:**
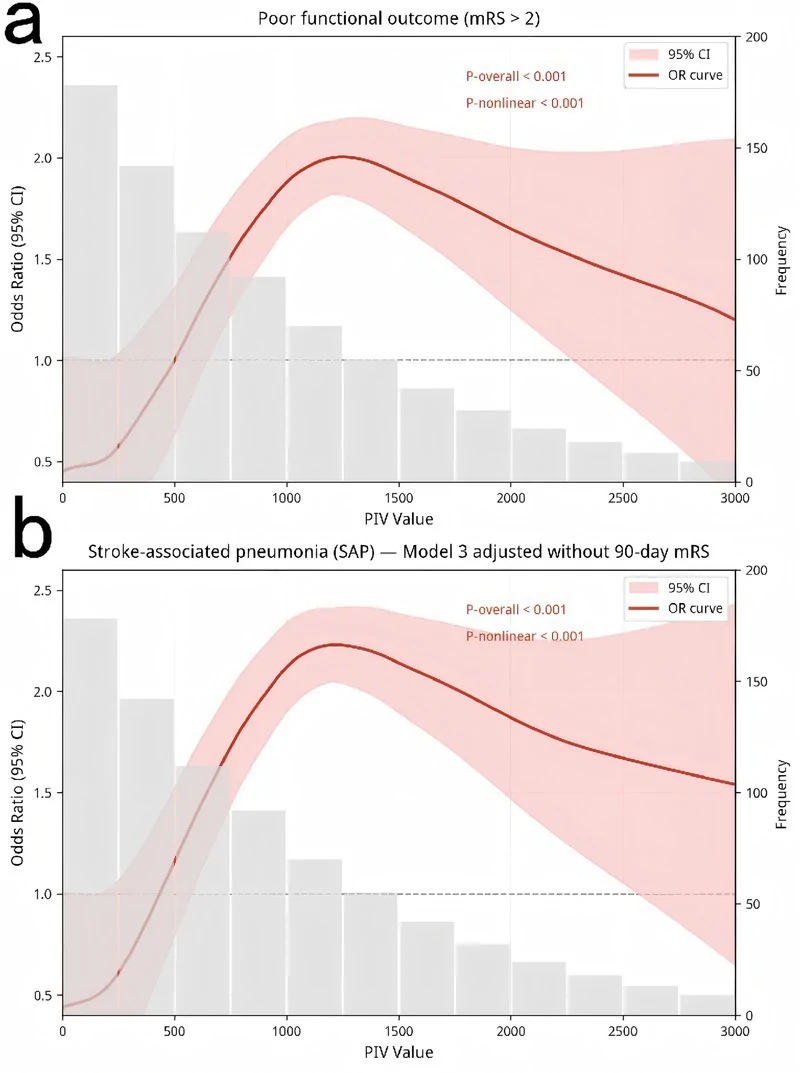
Nonlinear associations of PIV with SAP and poor functional outcome evaluated by restricted cubic spline analysis. **(a)** Restricted cubic spline plot illustrating the association between variables and poor functional outcome in patients with spontaneous intracerebral hemorrhage. **(b)** Restricted cubic spline plot illustrating the association between variables and SAP in patients with spontaneous intracerebral hemorrhage.

The predictive utility of PIV in forecasting SAP and poor functional outcome among patients with spontaneous intracerebral hemorrhage was assessed using subgroup and interaction analyses. During the analysis of SAP, PIV emerged as a significant predictor across various subgroups, including sex, age, hypertension, diabetes, GCS, EN, IVH, and hematoma location. In examining poor functional outcome, PIV consistently correlated with such outcomes in patients with spontaneous intracerebral hemorrhage across multiple subgroups, including sex, age, hypertension, diabetes, hyperlipidemia, SAP, the utilization of antiplatelet and anticoagulant medications, and GCS. Furthermore, the interaction analysis indicated that PIV’s predictive ability remained relatively stable, with no significant interaction effects observed among the groups (*p* for interaction > 0.05) (Figure 2).

**Figure 2:**
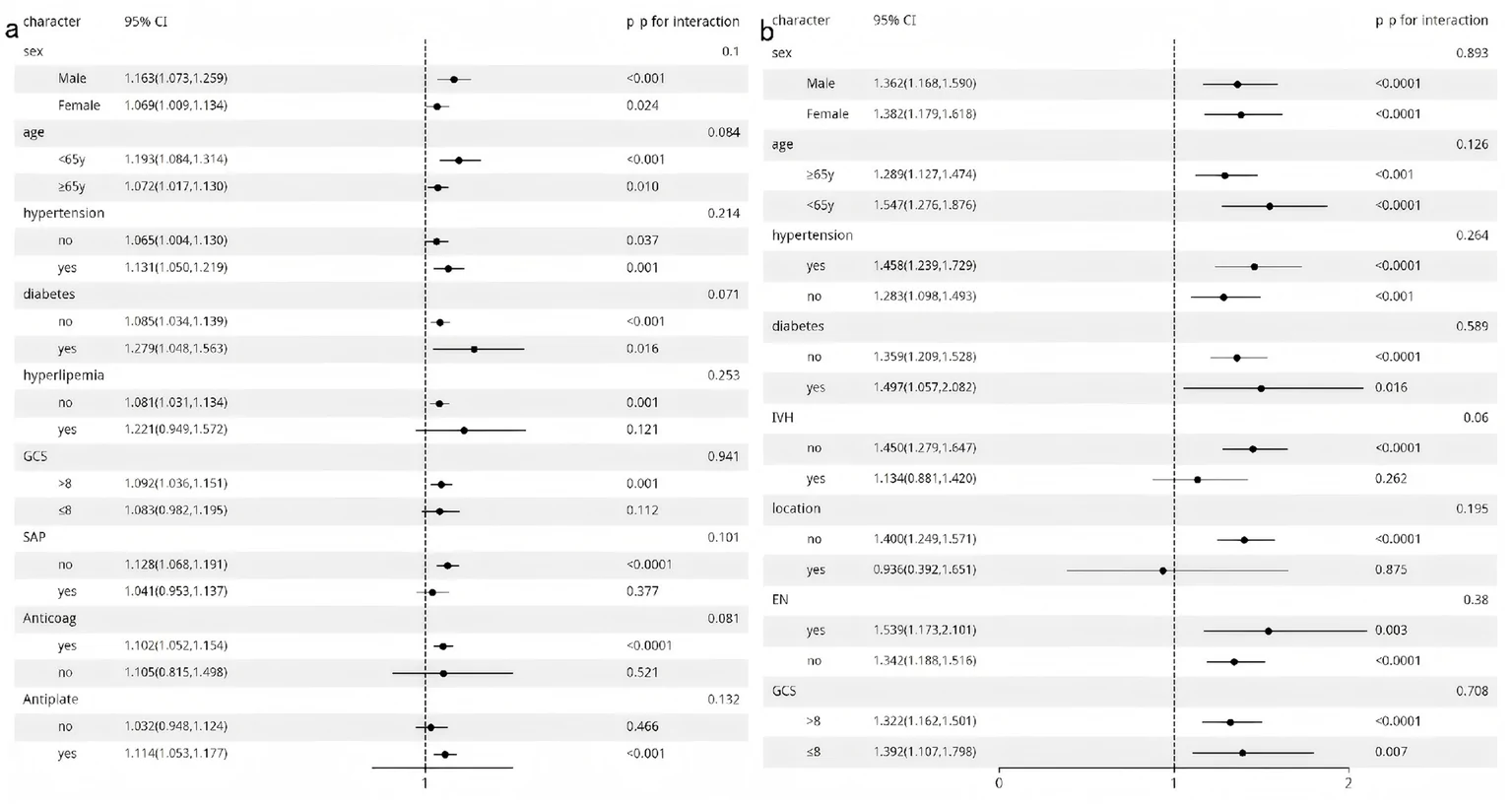
The predictive value of PIV in forecasting SAP and poor functional outcome in patients with spontaneous intracerebral hemorrhage was evaluated through subgroup and interaction analyses. **(a)** The predictive value of PIV in forecasting poor functional outcome in patients with spontaneous intracerebral hemorrhage was evaluated through subgroup and interaction analyses. **(b)** The predictive value of PIV in forecasting SAP in patients with spontaneous intracerebral hemorrhage was evaluated through subgroup and interaction analyses. Anticoag, anticoagulation; IVH, intraventricular hemorrhage; EN, enteral nutrition; location: hematoma location, whether it was supratentorial hemorrhage.

ROC curve analysis comparing the three prediction models (PIV, ICH-APS-A, and their combination) for SAP in spontaneous intracerebral hemorrhage patients showed that the combined model had the highest discrimination, with an AUC of 0.886. This was significantly greater than the AUC achieved by the PIV model (0.753) or the ICH-APS-A model (0.844) alone. The combined model demonstrated superior discriminatory ability, with an AUC of 0.886, outperforming both the PIV model (AUC: 0.753) and the ICH-APS-A model (AUC: 0.844) individually. The multivariate ROC comparison curves for predicting poor functional outcome in patients with spontaneous intracerebral hemorrhage using four composite inflammation indices—PIV, NLR, MLR, and PLR—revealed that PIV exhibited the best predictive performance (AUC: PIV 0.876, NLR 0.81, MLR 0.813, PLR 0.727) (Figure 3). Furthermore, the DCA curve indicated that the predictive model combining PIV and ICH-APS-A scores demonstrated superior clinical practical performance in predicting SAP in patients with spontaneous intracerebral hemorrhage compared to models that included only a single indicator. Additionally, the predictive model incorporating PIV showed better practical predictive performance for poor functional outcome in patients with spontaneous intracerebral hemorrhage compared to models that included other inflammation indices (NLR, MLR, PLR) (Figure 4).

**Figure 3:**
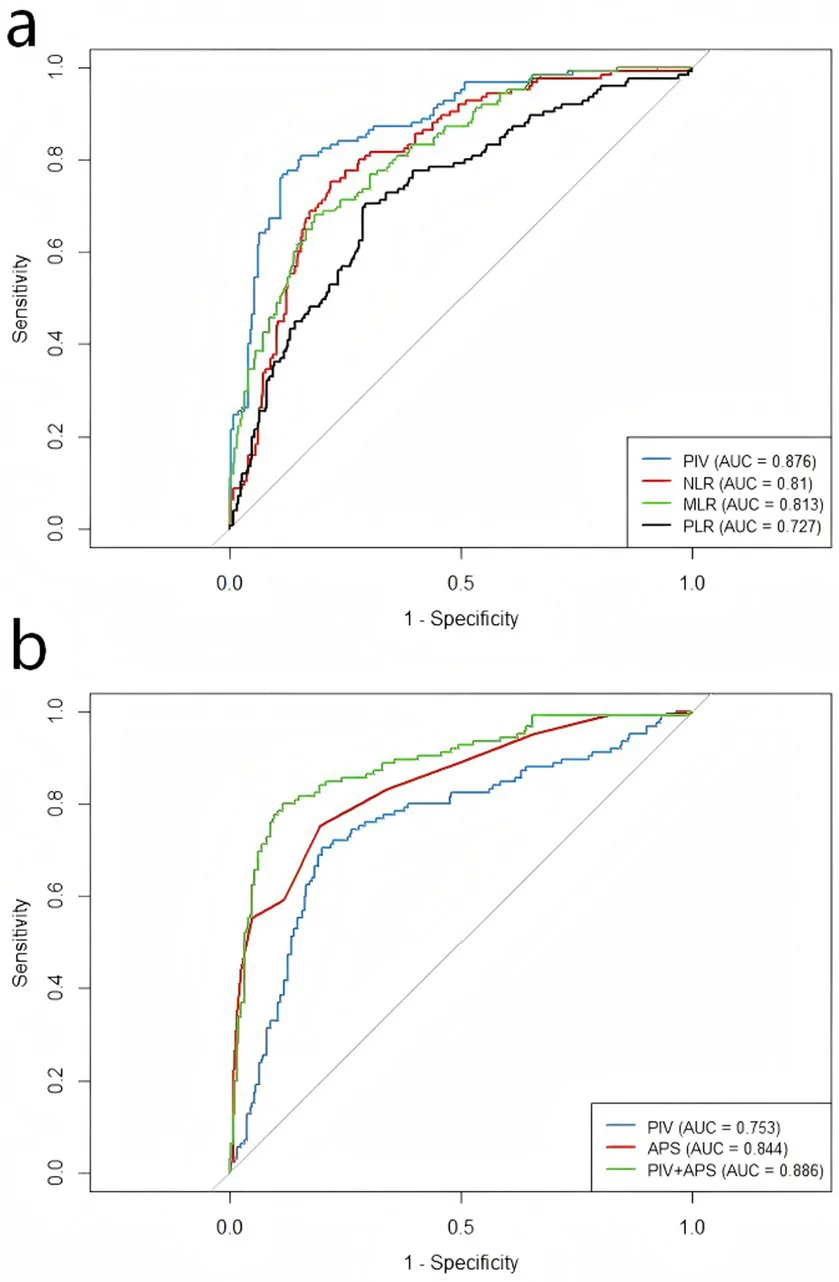
Multivariate receiver operating characteristic (ROC) curves. **(a)** Multivariate receiver operating characteristic (ROC) curves were constructed to evaluate and compare the predictive performance of four composite inflammation indices for poor functional outcome in patients with spontaneous intracerebral hemorrhage. **(b)** Multivariate receiver operating characteristic (ROC) curves were constructed to compare the predictive performance of baseline PIV, the Intracerebral Hemorrhage-Associated Pneumonia Prediction Scale A (ICH-APS-A), and their combined model in forecasting SAP among patients with spontaneous intracerebral hemorrhage. APS, ICH-APS-A scale; NLR, ratio of neutrophil count to lymphocytes count; MLR, ratio of mononuclear count to lymphocytes count; PLR, ratio of platelet count to lymphocytes count.

**Figure 4:**
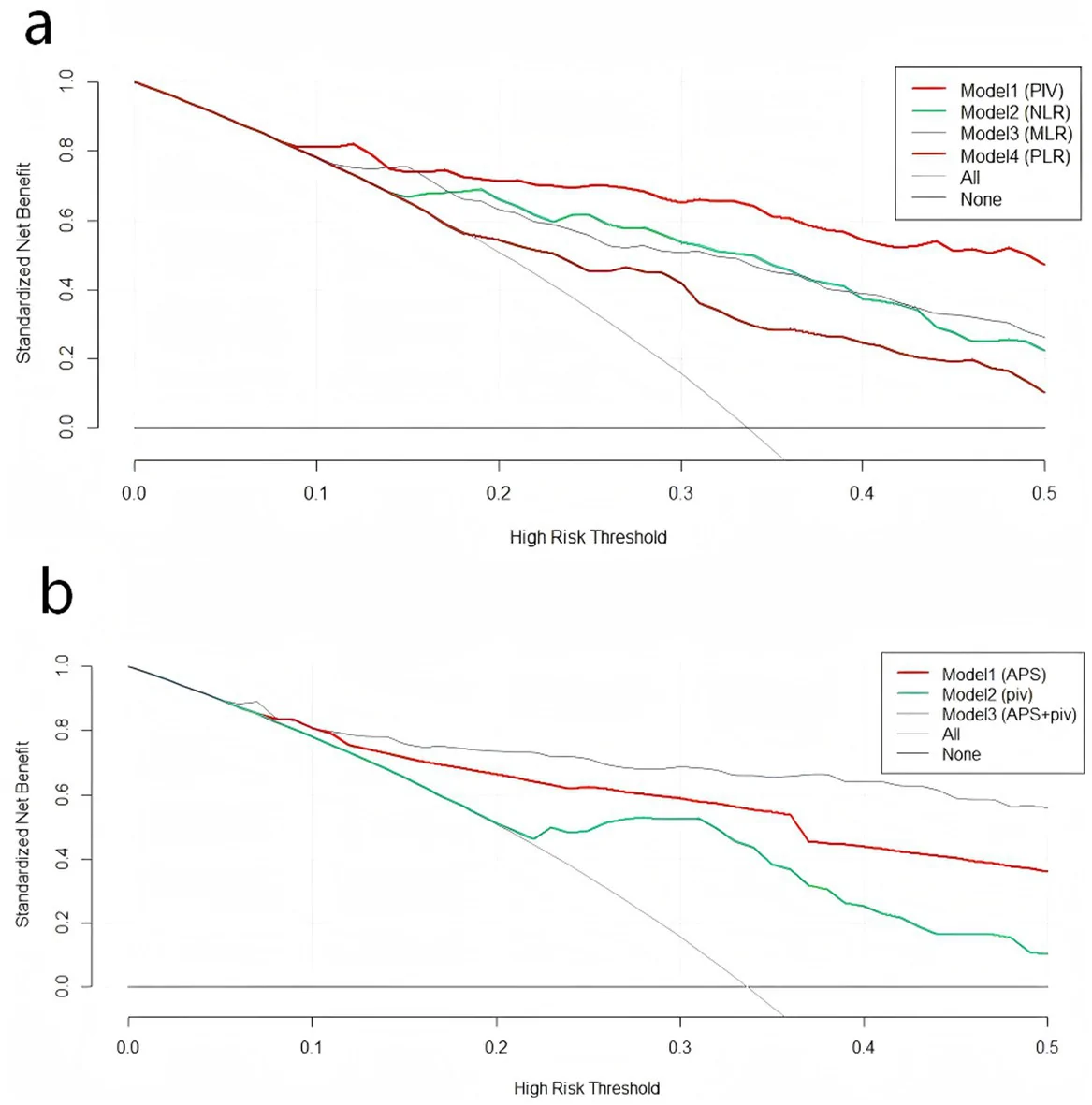
Multivariate decision curve analysis (DCA) curve. **(a)** DCA clinical decision curves were constructed to systematically evaluate and compare the predictive performance of four composite inflammation indices in forecasting poor functional outcome among patients with spontaneous intracerebral hemorrhage. **(b)** DCA clinical decision curves were constructed to compare the predictive performance of baseline PIV, the Intracerebral Hemorrhage-Associated Pneumonia Prediction Scale A (ICH-APS-A), and their integrated model in predicting SAP among patients with spontaneous intracerebral hemorrhage. APS ICH-APS-A scale; NLR, ratio of neutrophil count to lymphocytes count; MLR, ratio of mononuclear count to lymphocytes count; PLR, ratio of platelet count to lymphocytes count.

This study visualized the strength of correlations among feature variables by plotting a confusion matrix (Figure 5). Feature selection was performed using LASSO regression. After computation, seven variables—hematoma volume, NIHSS score, IVH, age, WBC, hemorrhage location, and sex—were retained to construct a binary predictive model for poor functional outcome(mRS > 2); eight variables—ICH-APS-A score, fibration, SEX, WBC, volume, Mg2+, APOB, and diabetes—were retained to construct a binary predictive model for SAP (Figure 6).

**Figure 5:**
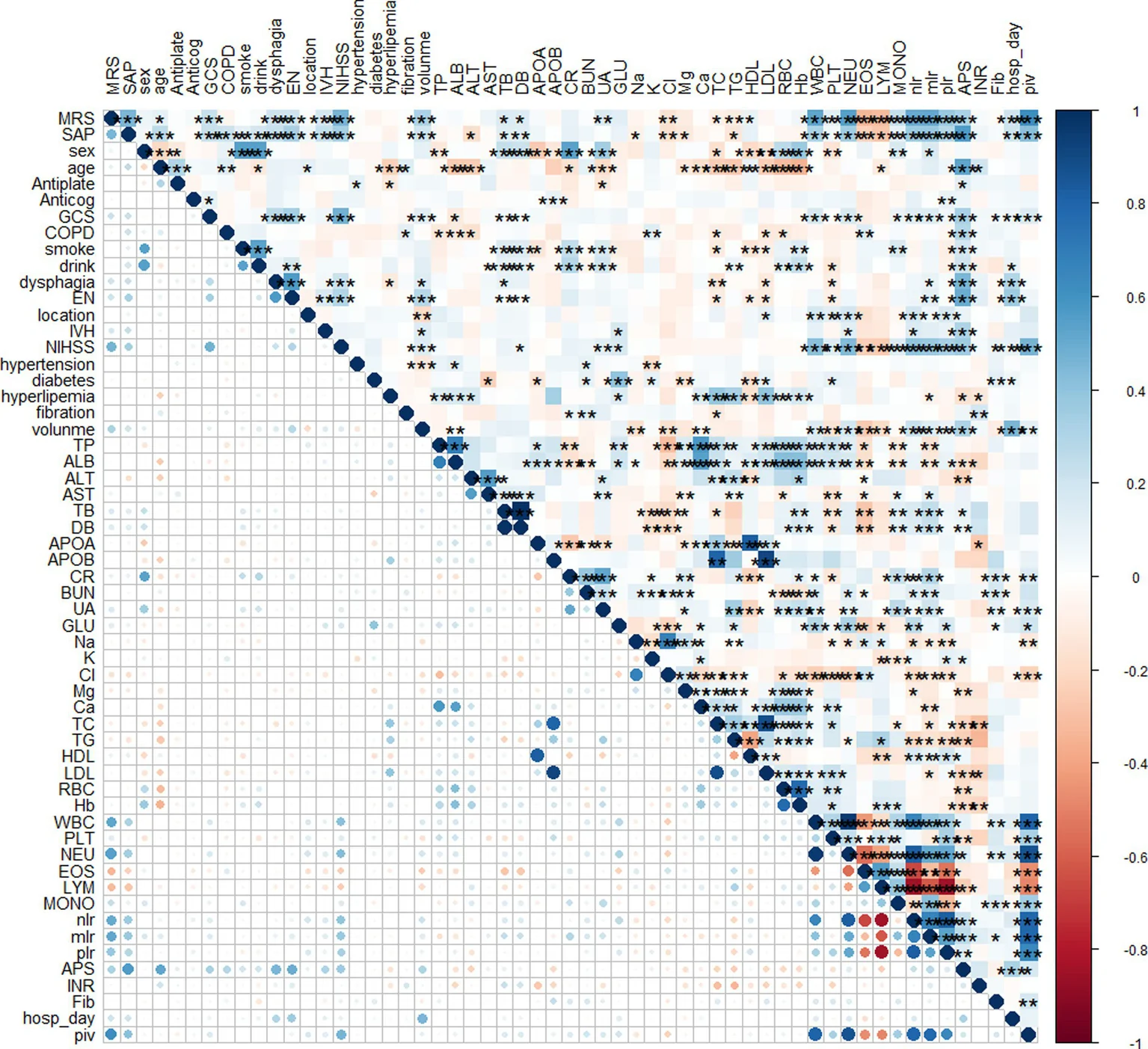
The confusion matrix of correlation between characteristic variables of the entire data set. Anticog, anticogulation; ACS, acute coronary syndrome; COPD, chronic obstructive pulmonary disease; GCS, Glasgow Coma Scale at baseline; NIHSS, National Institutes of Health Stroke Scale at baseline; mRS, Day 90 Modified Rankin Scale; EN, enteral nutrition; location, hematoma location, whether it was supratentorial hemorrhage; IVH, intraventricular hemorrhage; APS: ICH-APS-A Scale; volume, volume of hematoma (infratentorial hemorrhage volume ≥10 mL or supratentorial hemorrhage volume ≥40 mL); PIV, pan-immune inflammation value; nlr, ratio of neutrophil count to lymphocytes count; mlr, ratio of mononuclear count to lymphocytes count; plr, ratio of platelet count to lymphocytes count; WBC, White blood cell; PLT, platelet count; NEU, neutrophil count; EOS, eosinophils; LYM, lymphocyte count; MONO, monocyte count; RBC, red blood cell; Hb, hemoglobin; Fib, fibrinogen; TP, total protein; ALB, albumin; INR, international normalized ratio; ALT, alanine transaminase; AST, aspartate transaminase; TBIL, total bilirubin; IBIL, indirect bilirubin; CR, creatinine; BUN, blood urea nitrogen; UA, uric acid; GLU, glucose; Na, sodium; K, potassium; Cl, chloride; Mg, magnesium; Ca, calcium; TC, total cholesterol; TG, triglycerides; HDL, high density lipoprotein; LDL, low density lipoprotein; ApoA, apolipoprotein A; ApoB, apolipoprotein B; hosp_day, length of hospital stay for enrolled patients.

**Figure 6:**
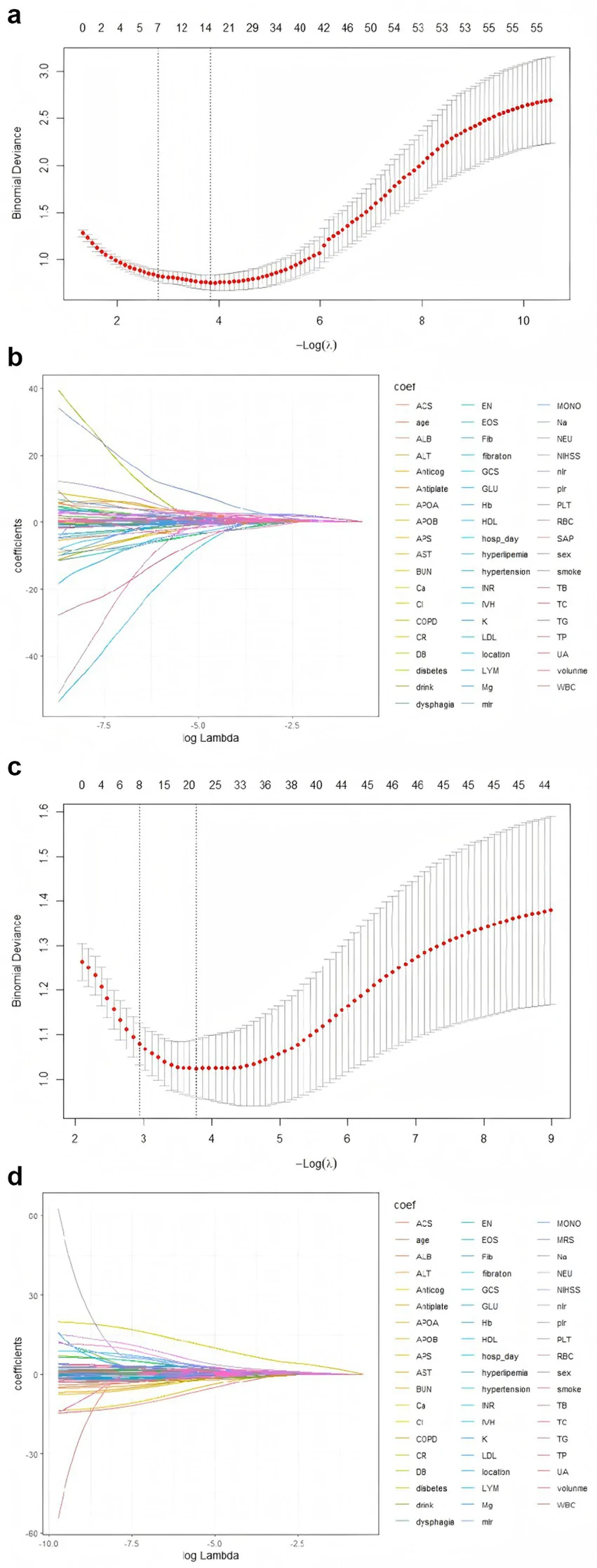
The LASSO regression coefficient path and cross-validation for feature selection. LASSO regression feature selection using poor functional outcome as the binary outcome: **(a)** LASSO regression cross-validation plot; **(b)** LASSO regression coefficient path plot. LASSO regression feature selection with SAP as the binary outcome variable. **(c)** LASSO regression cross-validation diagram; **(d)** LASSO regression coefficient path diagram.

Nine models were developed to predict poor functional outcome and SAP in patients with spontaneous intracerebral hemorrhage: XGBoost, LR, SVM, KNN, NB, DT, RF, AdaBoost, and GBM. Model performance was compared using ROC curves, with AUC as the primary metric. As shown in Figure 7, Table 3, among the nine models predicting poor functional outcome (mRS > 2), XGBoost performed best (AUC: 0.912, 95% CI: 0.855–0.969; accuracy: 0.827; sensitivity: 0.833; specificity: 0.823; F1 score: 0.808). As shown in Figure 7, Table 3, among the nine models predicting SAP, LR performed best (AUC: 0.856, 95% CI: 0.788–0.923, *p* < 0.05; accuracy: 0.774; sensitivity: 0.611; specificity: 0.853; F1 score: 0.637). Consequently, the XGBoost and LR models were selected for further investigation.

**Figure 7:**
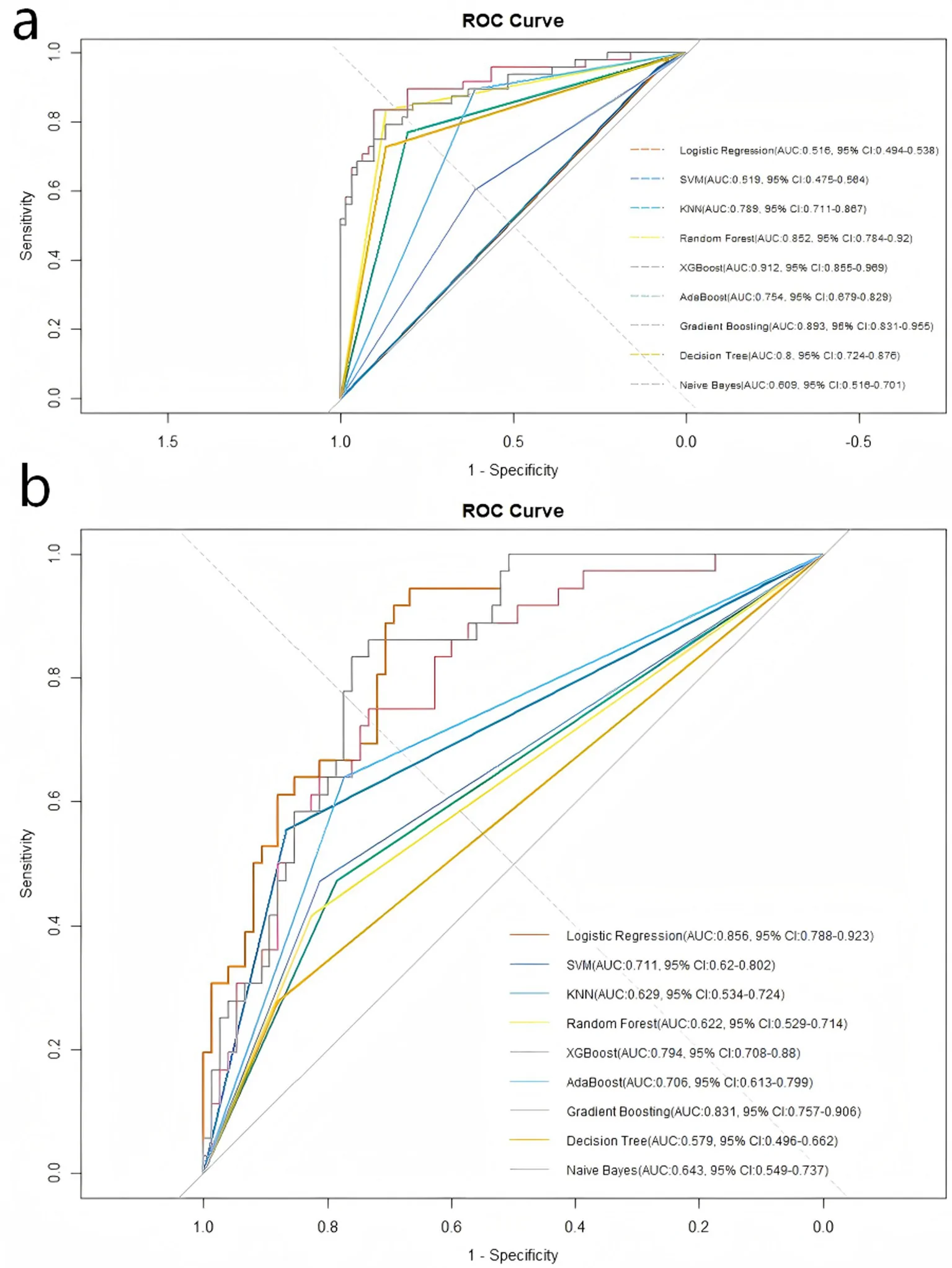
Comparison of Receiver Operating Characteristic (ROC) curves for nine machine learning models: **(a)** The representative nine machine learning models constructed with poor functional outcome as the binary outcome; **(b)** The representative nine machine learning models were constructed with SAP as the binary outcome.

**Table 3:** Performance comparison of nine machine learning models.

Model	AUC	Accuracy	Sensitivity	Specificity	PPV	NPV	F1 score
XGBoost	0.912298387	0.827272727	0.833333333	0.822580645	0.784313725	0.86440678	0.808080808
LR	0.79469086	0.454545455	1	0.032258065	0.444444444	1	0.615384615
SVM	0.519489247	0.463636364	0.958333333	0.080645161	0.446601942	0.714285714	0.609271523
KNN	0.788642473	0.790909091	0.770833333	0.806451613	0.755102041	0.819672131	0.762886598
NB	0.608534946	0.609090909	0.604166667	0.612903226	0.547169811	0.666666667	0.574257426
DT	0.800067204	0.809090909	0.729166667	0.870967742	0.813953488	0.805970149	0.769230769
RF	0.852150538	0.854545455	0.833333333	0.870967742	0.833333333	0.870967742	0.833333333
AdaBoost	0.75436828	0.736363636	0.895833333	0.612903226	0.641791045	0.88372093	0.747826087
GBM	0.893145161	0.8	0.854166667	0.758064516	0.732142857	0.87037037	0.788461538
XGBoost	0.794074074	0.747747748	0.611111111	0.813333333	0.611111111	0.813333333	0.611111111
LR	0.855555556	0.774774775	0.611111111	0.853333333	0.666666667	0.820512821	0.637681159
SVM	0.711111111	0.765765766	0.555555556	0.866666667	0.666666667	0.802469136	0.606060606
KNN	0.629444444	0.684684685	0.472222222	0.786666667	0.515151515	0.756410256	0.492753623
NB	0.642777778	0.702702703	0.472222222	0.813333333	0.548387097	0.7625	0.507462687
DT	0.578888889	0.684684685	0.277777778	0.88	0.526315789	0.717391304	0.363636364
RF	0.621666667	0.693693694	0.416666667	0.826666667	0.535714286	0.746987952	0.46875
AdaBoost	0.706111111	0.72972973	0.638888889	0.773333333	0.575	0.816901408	0.605263158
GBM	0.831111111	0.756756757	0.555555556	0.853333333	0.64516129	0.8	0.597014925

AUC: model discrimination; Accuracy: prediction correctness; Sensitivity: positive-class identification; Specificity: negative-class identification; PPV, positive predictive value; NPV, negative predictive value; F1 score: Harmonic mean of precision and recall.

After establishing the initial predictive models, “PIV” was incorporated to compare model performance before and after inclusion. As shown in Figure 8, the predictive performance of both models improved after incorporating PIV, with the AUC increasing from 0.926 to 0.942 for XGBoost and from 0.905 to 0.931 for logistic regression (*p* < 0.05). DeLong tests further confirmed that these differences were statistically significant (both *p* < 0.05). Calibration curves and the H–L test indicated that the goodness-of-fit of the models improved to some extent after incorporating “PIV” (Figure 9). Similarly, DCA demonstrated that the models including “PIV” achieved greater clinical benefit compared with those without “PIV” (Figure 10).

**Figure 8:**
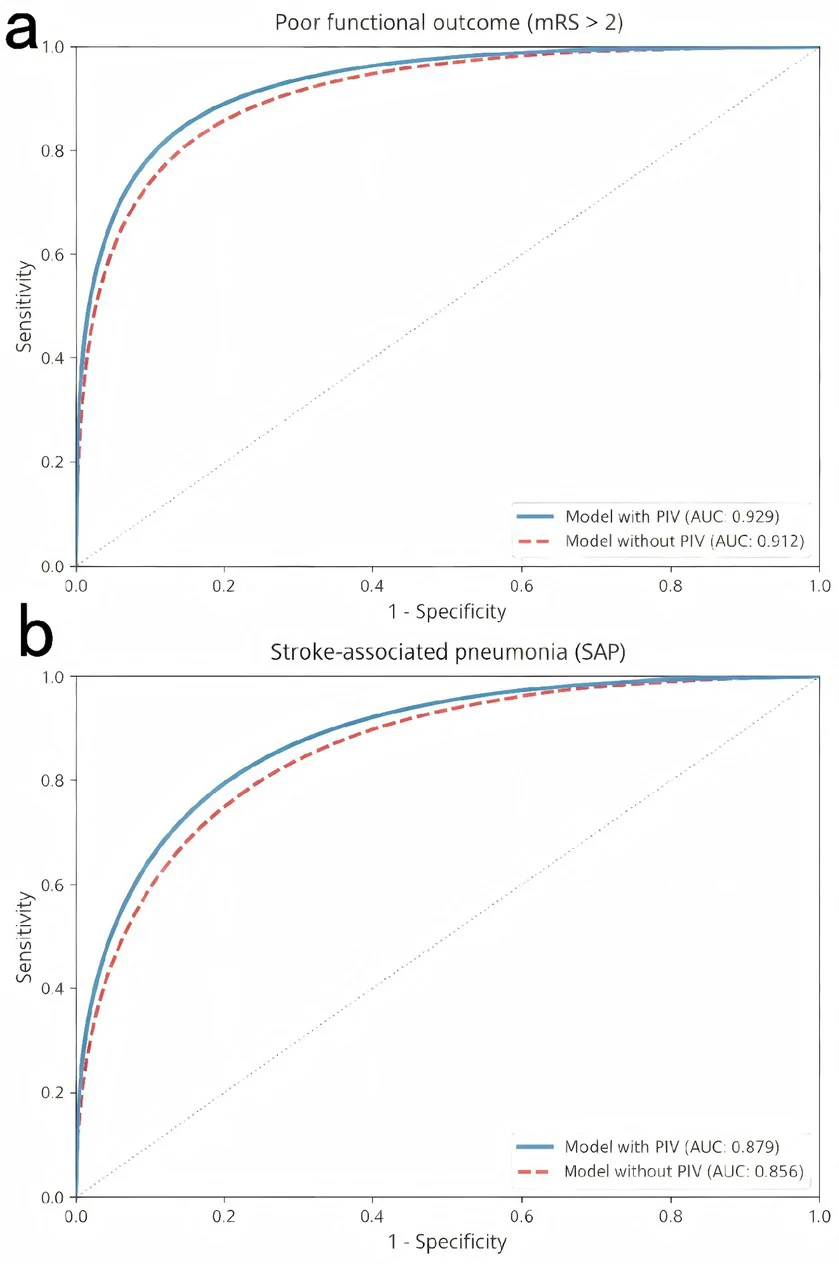
ROC curve comparison before and after integration of “PIV.” **(a)** ROC curve variation of clinical prediction model constructed with poor functional outcome as binary outcome before and after integration of “PIV.” **(b)** ROC curve variation of clinical prediction model constructed with SAP as binary outcome before and after integration of “PIV”.

**Figure 9:**
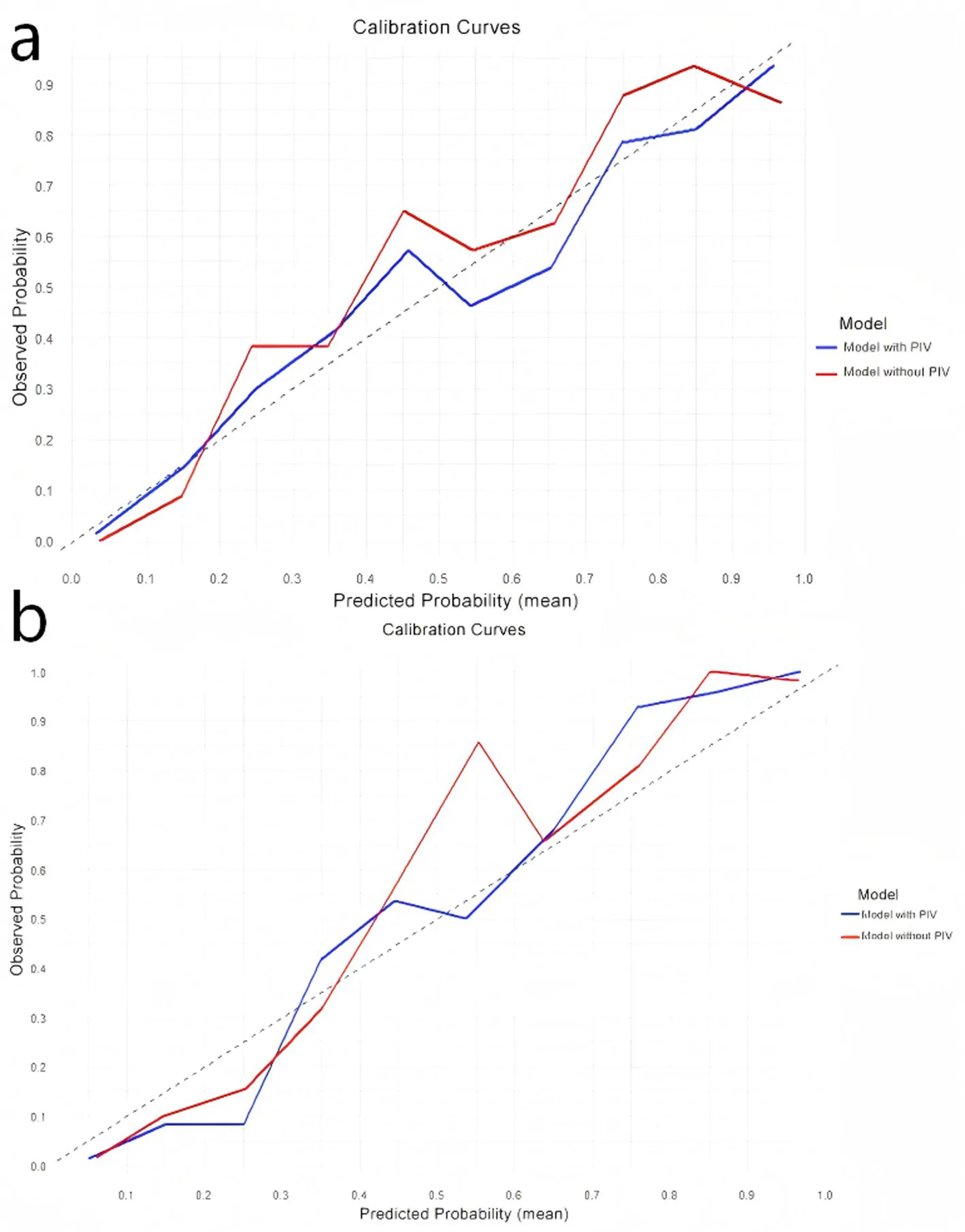
Comparison of calibration curves before and after integration of “PIV.” **(a)** Changes in the calibration curve of the clinical prediction model with poor functional outcome as a binary outcome before and after integration of “PIV.” **(b)** Changes in the calibration curve of the clinical prediction model with SAP as a binary outcome before and after integration of “PIV”.

**Figure 10:**
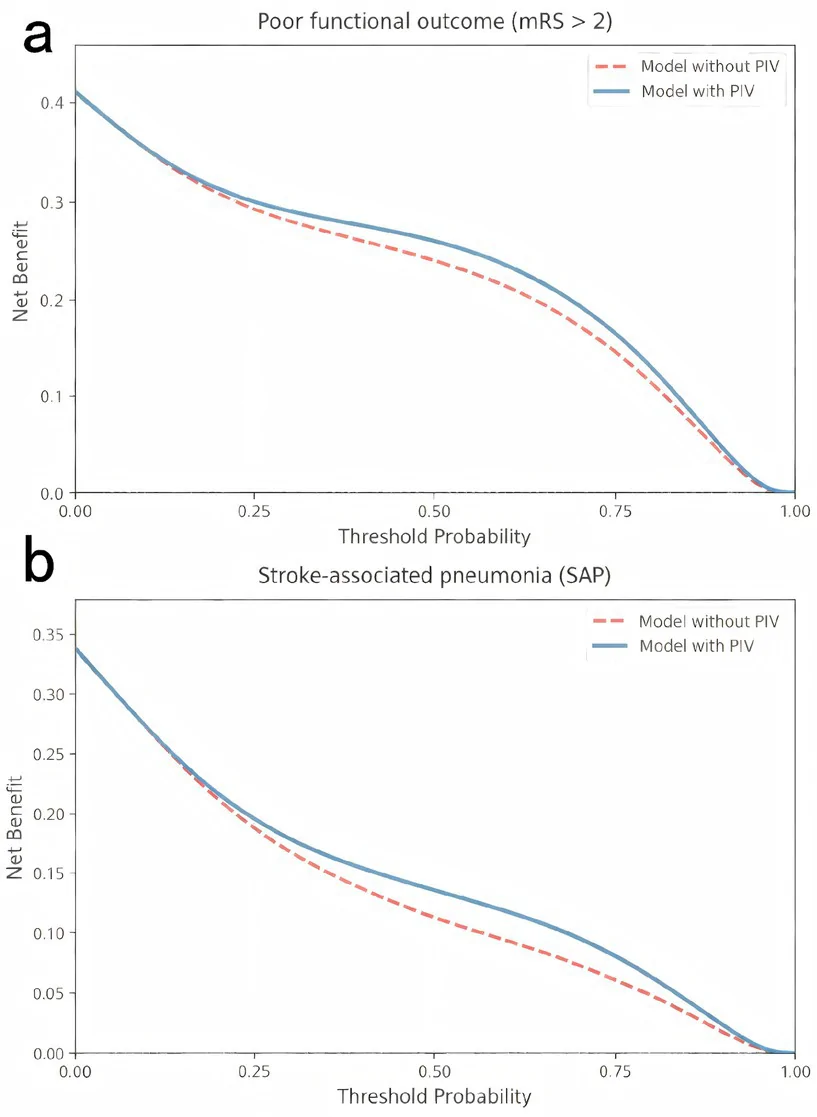
Comparison of decision curve analysis (DCA) before and after integration of “PIV.” **(a)** The changes of DCA curves before and after “PIV” integration in clinical prediction models constructed with poor functional outcome as binary outcomes. **(b)** The changes of DCA curves before and after “PIV” in clinical prediction models constructed with SAP as a binary outcome.

SHAP analysis was performed to interpret the feature importance in two predictive models. In the model for 90-day poor functional outcome (mRS > 2), the PIV demonstrated a mean absolute SHA*p* value of 0.312, which was comparable to that of age (0.344) and exceeded values for white blood cell count (0.238) and hematoma location (0.213). SHAP dependence plots indicated a positive association between higher PIV and increased risk of poor functional outcome (Figures 11a,b); in an exemplary case, a patient with PIV = 0.587 received a SHAP contribution of +0.587, identifying PIV as a primary risk driver (Figures 11c,d). In the prediction model for stroke-associated pneumonia (SAP), the pan-immune-inflammation value (PIV) ranked third in importance, with a mean absolute SHA*p* value of 0.368, following only the APS (0.482) and volume (0.433). It exceeded conventional indicators such as APOB (0.367), white blood cell count (0.346), and magnesium level (0.150). Consistently, higher PIV values were associated with an increased predicted risk of pneumonia in dependency plots (Figures 12a,b). In a representative patient with a PIV of 0.347, PIV contributed +1.15 to the risk score (Figures 12c,d). Collectively, these analyses establish PIV as a robust and independent predictor in this prognostic context, providing added value over traditional inflammatory and clinical markers.

**Figure 11:**
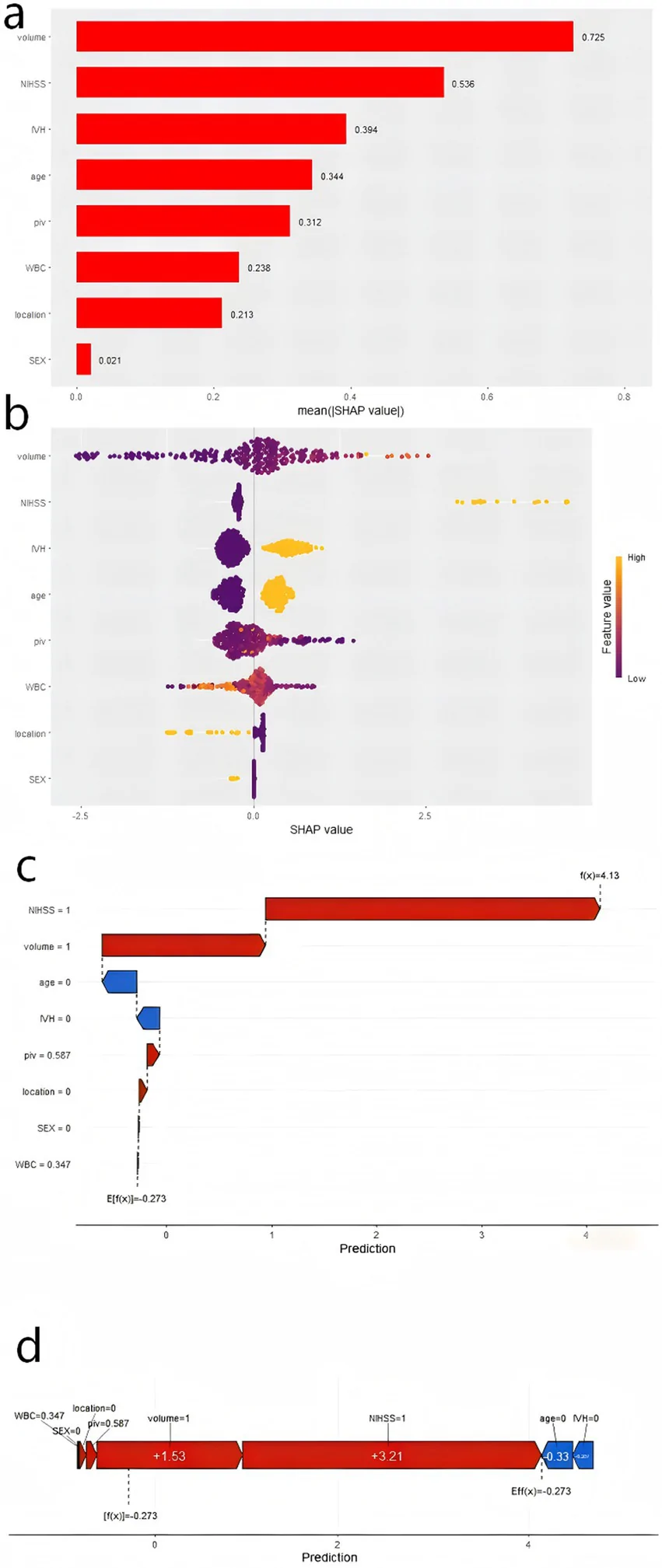
Global and local model explanation by the SHAP method. Global and local SHAP interpretation of the final clinical prediction model constructed with poor functional outcome as binary outcomes. **(a)** SHAP summary bar plot. This plot evaluates the contribution of each feature to the model using mean SHAP values, displayed in descending order. **(b)** SHAP summary dot plot. **(c)** SHAP waterfall plot. This plot shows the contribution of each feature to the prediction result of one patient using the XGBoost model. Red bars indicate features that contribute positively to the prediction, while blue bars indicate negative contributions. **(d)** SHAP force plot.

**Figure 12:**
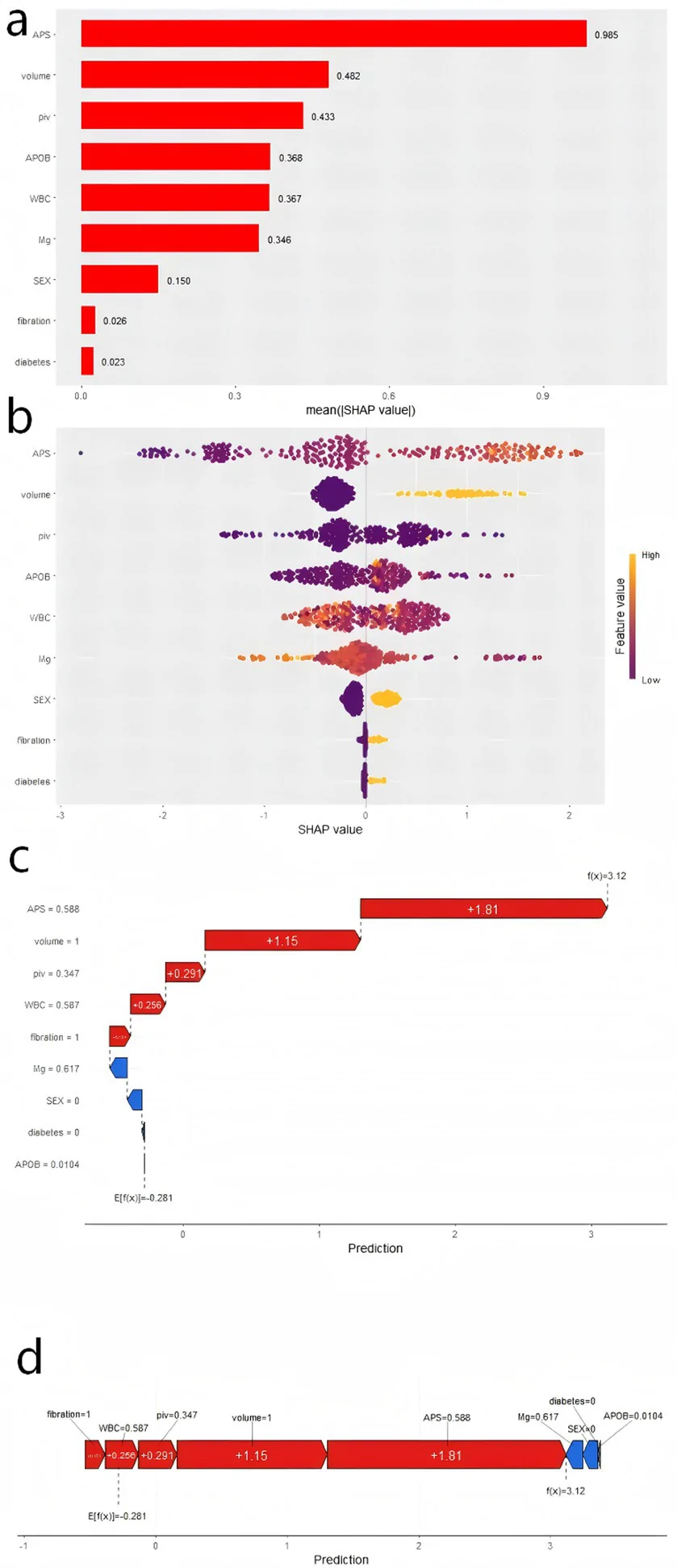
Global and local model explanation by the SHAP method. Global and local SHAP interpretation of the final clinical prediction model constructed with SAP as a binary outcome. **(a)** SHAP summary bar plot. This plot evaluates the contribution of each feature to the model using mean SHAP values, displayed in descending order. **(b)** SHAP summary dot plot. **(c)** SHAP waterfall plot. This plot shows the contribution of each feature to the prediction result of one patient using the LR model. Red bars indicate features that contribute positively to the prediction, while blue bars indicate negative contributions. **(d)** SHAP force plot.

## Discussion

This study tested the core hypothesis that integrating PIV into ML models would significantly enhance the prediction of SAP and 90-day poor functional outcomes in patients with sICH. The results confirmed this hypothesis, demonstrating that PIV is an independent and robust predictor of both endpoints (SAP: OR 13.55, 95% CI 4.14–44.39; *p* < 0.0001; poor functional outcome: OR 25.27, 95% CI 7.75–82.34; *p* < 0.0001). Furthermore, incorporating PIV into the optimal ML models (XGBoost for poor functional outcomes and LR for SAP) yielded significant improvements in predictive performance, with enhanced discrimination, calibration, and clinical utility confirmed by ROC analysis, calibration curves, and DCA. Taken together, these findings suggest that PIV, as a comprehensive marker of systemic immune-inflammatory status, captures unique pathophysiological information that complements traditional clinical, imaging, and laboratory variables. These observations provide a foundation for further investigation and external validation toward eventual personalized risk stratification in neurocritical care.

PIV is a composite index integrating neutrophils, monocytes, platelets, and lymphocyte counts, providing a holistic reflection of systemic inflammatory and immune status (11, 12), with demonstrated prognostic value across various diseases (27–34). Although PIV has shown utility in other cerebrovascular contexts, its specific role in predicting SAP and functional recovery after spontaneous sICH remained unexplored. This study directly addresses this gap, systematically demonstrating PIV’s superior discriminative performance against established ratios like NLR, PLR, and MLR for 90-day poor functional outcome (AUC: PIV 0.876; NLR 0.810; MLR 0.813; PLR 0.727) (5, 35, 36) and its incremental value when combined with the clinical ICH-APS-A score (19) for SAP prediction (combined AUC: 0.886).

This enhanced performance originates from PIV’s integrative design, which simultaneously captures four key cellular players in the immune-thrombotic network: neutrophils, monocytes, platelets, and lymphocytes. Neutrophils represent innate immune activation; monocytes amplify inflammation via cytokine release; platelets contribute to thrombo-inflammation and blood–brain barrier disruption; and lymphocytes reflect adaptive immune dysfunction, a hallmark of secondary injury after sICH (37–44). By synthesizing these interconnected pathways, PIV provides a comprehensive measure of the systemic immune dysregulation that underlies both SAP and unfavorable recovery.

The combined PIV + ICH-APS-A model effectively leverages these insights, achieving superior SAP prediction (AUC 0.886) compared to either marker alone (ICH-APS-A:0.844; PIV: 0.753). This synergy stems from complementary information: ICH-APS-A quantifies initial structural damage (19), while PIV captures the dynamic immune-inflammatory response. Together, they offer a more precise tool for risk stratification in neurocritical care—a notable advance in a setting where inaccurate SAP prediction often leads to unwarranted antibiotic prophylaxis (7, 8).

Beyond its role in synergistic prediction models, the intrinsic prognostic value of PIV was further elucidated through dose–response analysis. Restricted cubic spline curves revealed a significant nonlinear relationship between PIV and the risk of both SAP and poor functional outcome (*p*-nonlinear <0.001). The risk for each endpoint exhibited a steady increase across the PIV range, with a notably steeper rise beyond approximately 1,500–2000, confirming its utility as a continuous and progressive risk indicator. Subgroup analyses demonstrated internal consistency of the PIV-outcome association across key clinical strata within this cohort. No significant interaction effects were observed across key clinical strata, including sex, age, hypertension, diabetes, baseline GCS, and the presence of IVH (all *p* for interaction >0.05), for either outcome. However, we acknowledge that these analyses are exploratory, and the relatively small sample sizes in certain subgroups preclude definitive conclusions about generalizability. Independent replication in larger cohorts is required to confirm these internal observations.

The predictive value of PIV was further examined within a comprehensive machine learning framework. XGBoost is adept at modeling the complex, nonlinear interactions relevant to long-term recovery, whereas logistic regression offers the interpretability necessary for a clinically translatable SAP risk tool. To elucidate the mechanics underlying the optimized models, SHAP analysis was employed to quantify feature importance. This interpretability technique identified PIV as a consistently influential contributor, ranking among the top five features in both predictive models. More importantly, SHAP dependence plots consistently revealed a positive, monotonic relationship between higher PIV values and an increased predicted risk of adverse outcomes. This data-driven pattern positions PIV not merely as a correlative marker, but as an active component within the prognostic logic of the models, directly linking the degree of systemic inflammation to the probability of clinical deterioration. The convergence of its strong SHAP ranking and consistent directional effect solidifies its role as a core element in the data-driven prognostic assessment of sICH.

While these findings highlight the prognostic value of PIV, this study has several important limitations that must be acknowledged. Most critically, its retrospective, single-center design and the absence of an external validation cohort severely limit the generalizability of the predictive models. The reported performance metrics (e.g., AUC) are likely optimistic and may not generalize to other populations. The models presented here should be considered internally developed prototypes requiring independent validation before any clinical application can be contemplated. Second, the retrospective nature may lead to residual confounding despite statistical adjustments. Third, the reliance on subjective GCS and NIHSS scores might introduce measurement bias. Fourth, only baseline PIV was assessed without tracking its dynamic changes during disease progression. Finally, the research focused solely on hemorrhagic stroke, leaving PIV’s predictive value in other stroke types unexplored. Future research should address these limitations by: (1) conducting prospective multi-center studies with external validation to confirm generalizability; (2) incorporating dynamic PIV monitoring to evaluate serial measurements’ predictive value; (3) operationalizing the optimally performing models into clinically deployable tools, such as simplified risk scores or mobile health applications, contingent upon successful external validation.

## Conclusion

In summary, this study suggests that integrating PIV into ML models may improve the prediction of SAP and 90-day poor functional outcomes in spontaneous sICH. PIV appears to be superior to traditional inflammatory markers in this context, likely due to its comprehensive assessment of the immune-thrombotic network. Its consistent predictive value across subgroups demonstrates internal consistency within this cohort. The optimal models identified (XGBoost for poor functional outcomes, LR for SAP) provide a balance between performance and interpretability, which may facilitate future clinical translation. However, these findings should be considered preliminary, and the models are not yet ready for routine clinical use. With prospective external validation, PIV-integrated models could eventually become a valuable tool for identifying high-risk patients, guiding targeted interventions, and improving outcomes in sICH.

## Data Availability

The datasets generated and/or analyzed during the current study were collected at Longyan First Affiliated Hospital of Fujian Medical University and are not publicly available due to patient privacy and ethical restrictions. De-identified data may be available to qualified researchers upon reasonable request, subject to appropriate ethical approvals and the execution of a data use agreement. Requests should be directed to: Xiaohong Hu, Neurology Department, Longyan First Affiliated Hospital of Fujian Medical University, No. 105, Jiuyi North Road, Longyan, Fujian, China, 364000; Email: hxh19790601@sina.com; Cell phone: +86-138-5068-5180.
