# Supplementary material for: The pan-immune-inflammation value predicts stroke-associated pneumonia and poor outcome in spontaneous intracerebral hemorrhage: a machine learning approach
Source: Front Neurol. 2026 Sep 17;17:1906754. doi: 10.3389/fneur.2026.1906754 (PMC13627042; doi:10.3389/fneur.2026.1906754)
Supplement: Supplementary file 1 [file Table_1.docx]

**Supplementary Table 1** The baseline demographics and clinical attributes stratified by the occurrence of SAP groupings.

| **variable** | **Total**  **(n=371)** | **SAP**  **(n=125)** | **non-SAP**  **(n=246)** | **p.value** |
| --- | --- | --- | --- | --- |
| **sex** |  |  |  | 0.37 |
| female | 144(38.81) | 44(35.20) | 100(40.65) |  |
| male | 227(61.19) | 81(64.80) | 146(59.35) |  |
| **age** |  |  |  | **<0.0001** |
| ＜65 | 184(49.60) | 40(32.00) | 144(58.54) |  |
| ≥65 | 187(50.40) | 85(68.00) | 102(41.46) |  |
| **smoke** |  |  |  | **0.04** |
| no | 254(68.46) | 76(60.80) | 178(72.36) |  |
| yes | 117(31.54) | 49(39.20) | 68(27.64) |  |
| **drink** |  |  |  | **0.02** |
| no | 242(65.23) | 66(52.80) | 176(71.54) |  |
| yes | 129(34.77) | 59(47.20) | 70(28.46) |  |
| **Antiplate** |  |  |  | 0.99 |
| no | 337(90.84) | 113(90.40) | 224(91.06) |  |
| yes | 34( 9.16) | 12( 9.60) | 22( 8.94) |  |
| **Anticog** |  |  |  | 0.89 |
| no | 360(97.04) | 122(97.60) | 238(96.75) |  |
| yes | 11( 2.96) | 3( 2.40) | 8( 3.25) |  |
| **hypertension** |  |  |  | **0.03** |
| no | 54(14.56) | 19(15.20) | 35(14.23) |  |
| yes | 317(85.44) | 106(84.80) | 211(85.77) |  |
| **diabetes** |  |  |  | **<0.001** |
| no | 314(84.64) | 107(85.60) | 207(84.15) |  |
| yes | 57(15.36) | 18(14.40) | 39(15.85) |  |
| **ACS** |  |  |  | 1.00 |
| no | 353(95.15) | 119(95.20) | 234(95.12) |  |
| yes | 18( 4.85) | 6( 4.80) | 12( 4.88) |  |
| **hyperlipemia** |  |  |  | 0.23 |
| no | 217(58.49) | 79(63.20) | 138(56.10) |  |
| yes | 154(41.51) | 46(36.80) | 108(43.90) |  |
| **fibration** |  |  |  | **0.04** |
| no | 358(96.50) | 120(96.00) | 238(96.75) |  |
| yes | 13( 3.50) | 5( 4.00) | 8( 3.25) |  |
| **COPD** |  |  |  | **<0.0001** |
| no | 357(96.23) | 113(90.40) | 244(99.19) |  |
| yes | 14( 3.77) | 12( 9.60) | 2( 0.81) |  |
| **GCS** |  |  |  | **<0.0001** |
| ＞8 | 352(94.88) | 110(88.00) | 242(98.37) |  |
| ≤8 | 19( 5.12) | 15(12.00) | 4( 1.63) |  |
| **NIHSS** |  |  |  | **<0.0001** |
| ＜15 | 300(80.86) | 78(62.40) | 222(90.24) |  |
| ≥15 | 71(19.14) | 47(37.60) | 24( 9.76) |  |
| **mRS** |  |  |  | **<0.0001** |
| >2 | 125(33.69) | 80(64.00) | 45(18.29) |  |
| ≤2 | 246(66.31) | 45(36.00) | 201(81.71) |  |
| **dysphagia** |  |  |  | **<0.0001** |
| no | 312(84.10) | 91(72.80) | 221(89.84) |  |
| yes | 59(15.90) | 34(27.20) | 25(10.16) |  |
| **EN** |  |  |  | **<0.0001** |
| no | 275(74.12) | 68(54.40) | 207(84.15) |  |
| yes | 96(25.88) | 57(45.60) | 39(15.85) |  |
| **location** |  |  |  | **0.04** |
| no | 54(14.56) | 23(18.40) | 31(12.60) |  |
| yes | 317(85.44) | 102(81.60) | 215(87.40) |  |
| **IVH** |  |  |  | **<0.0001** |
| no | 229(61.73) | 57(45.60) | 172(69.92) |  |
| yes | 142(38.27) | 68(54.40) | 74(30.08) |  |
| **volunme** |  |  |  | **<0.01** |
| no | 319(85.98) | 79(63.2) | 240(97.56) |  |
| yes | 52(14.01) | 46(36.8) | 6( 2.43) |  |
| **APS** | 5.06 ± 3.36 | 7.81 ± 3.28 | 3.66 ± 2.41 | **<0.0001** |
| **PIV** | 464.83(241.66,883.85) | 880.01(554.28,1335.49) | 358.88(215.28,583.85) | **<0.0001** |
| **NLR** | 5.80(3.22,10.80) | 10.60(5.44,14.66) | 4.59(2.88,7.69) | **<0.0001** |
| **MLR** | 0.32(0.22,0.45) | 0.42(0.30,0.59) | 0.28(0.21,0.36) | **<0.0001** |
| **PLR** | 173.86(123.51,248.33) | 214.61(148.80,332.00) | 161.20(116.70,214.59) | **<0.0001** |
| **WBC** | 9.14(7.17,11.71) | 11.02(8.74,13.09) | 8.19(6.81,10.57) | **<0.0001** |
| **PLT** | 215.00(184.00,257.50) | 218.00(187.00,261.00) | 213.00(182.25,255.75) | **0.04** |
| **NEU** | 7.17(5.16,10.23) | 9.91(6.50,11.37) | 6.02(4.61,8.64) | **<0.0001** |
| **EOS** | 0.03(0.01,0.10) | 0.01(0.00,0.05) | 0.04(0.01,0.11) | **0.01** |
| **LYM** | 1.21(0.87,1.67) | 0.96(0.71,1.43) | 1.32(1.01,1.77) | **<0.0001** |
| **MONO** | 0.40(0.31,0.53) | 0.44(0.32,0.57) | 0.39(0.30,0.51) | **0.03** |
| **RBC** | 4.69 ± 0.65 | 4.67 ± 0.67 | 4.71 ± 0.65 | 0.58 |
| **Hb** | 139.75 ± 16.68 | 138.18 ± 16.86 | 140.54 ± 16.56 | 0.20 |
| **INR** | 1.02 ± 0.23 | 1.01 ± 0.11 | 1.02 ± 0.28 | 0.85 |
| **Fib** | 3.29 ± 0.90 | 3.32 ± 0.86 | 3.27 ± 0.92 | 0.63 |
| **TP** | 70.71 ± 5.53 | 70.39 ± 5.66 | 70.87 ± 5.47 | 0.43 |
| **ALB** | 41.32 ± 3.49 | 41.08 ± 3.63 | 41.44 ± 3.41 | 0.37 |
| **ALT** | 19.00(14.00,27.70) | 16.00(13.00,24.00) | 20.00(15.00,29.00) | 0.15 |
| **AST** | 24.00(21.00,30.00) | 24.00(20.00,30.00) | 24.00(21.00,31.00) | 0.29 |
| **TBIL** | 12.20(9.10,15.65) | 13.40(9.70,18.10) | 11.60(9.00,14.68) | 0.06 |
| **IBIL** | 9.70(7.30,12.60) | 10.90(7.40,14.20) | 9.25(7.23,11.78) | 0.06 |
| **CR** | 72.00(60.00,87.00) | 72.00(63.00,92.00) | 71.00(58.00,86.00) | 0.06 |
| **BUN** | 5.35(4.37,6.53) | 5.67(4.38,6.81) | 5.24(4.35,6.47) | 0.27 |
| **UA** | 341.00(278.50,422.00) | 342.00(281.00,423.00) | 338.00(278.25,419.75) | 0.54 |
| **GLU** | 7.14(6.07,8.66) | 7.32(6.19,8.93) | 7.02(5.96,8.48) | 0.25 |
| **Na** | 139.00(137.10,140.90) | 138.80(136.60,140.90) | 139.15(137.30,140.90) | 0.06 |
| **K** | 3.72 ± 0.41 | 3.76 ± 0.46 | 3.70 ± 0.39 | 0.19 |
| **Cl** | 103.30(101.00,105.45) | 102.60(99.90,104.80) | 103.70(101.50,105.68) | 0.06 |
| **Mg** | 0.82(0.76,0.87) | 0.80(0.75,0.86) | 0.82(0.77,0.87) | **0.02** |
| **Ca** | 2.29 ± 0.12 | 2.28 ± 0.13 | 2.30 ± 0.12 | 0.28 |
| **TC** | 5.09(4.32,5.85) | 5.03(4.30,5.76) | 5.09(4.33,5.87) | 0.33 |
| **TG** | 1.13(0.80,1.93) | 0.99(0.76,1.61) | 1.23(0.83,2.11) | 0.20 |
| **HDL** | 1.26(1.08,1.52) | 1.30(1.12,1.58) | 1.24(1.05,1.48) | 0.06 |
| **LDL** | 3.12(2.57,3.68) | 3.11(2.59,3.66) | 3.14(2.56,3.70) | 0.94 |
| **APOA** | 1.39(1.23,1.59) | 1.42(1.26,1.59) | 1.38(1.21,1.59) | 0.52 |
| **APOB** | 0.97(0.77,1.16) | 0.95(0.78,1.15) | 0.97(0.77,1.17) | **0.02** |
| **hosp_day** | 19.65 ± 19.42 | 24.03 ± 23.97 | 17.42 ± 16.25 | **<0.01** |

Anticog，Anticogulation；ACS，acute coronary syndrome；COPD, chronic obstructive pulmonary disease; GCS, Glasgow Coma Scale at baseline ; NIHSS，national institutes of health stroke scale at baseline；mRS，Day 90’Modified Rankin Scale；EN，enteral nutrition；location，hematoma location, whether it was supratentorial hemorrhage；IVH，intraventricular hemorrhage；APS，ICH-APS-A Scale；volunme, Volume of hematoma(Infratentorial hemorrhage volume ≥10 mL or Supratentorial hemorrhage volume ≥40 mL)；PIV, pan-immune inflammation value; NLR, Ratio of neutrophil count to lymphocytes count；MLR，Ratio of mononuclear count to lymphocytes count；PLR, Ratio of platelet count to lymphocytes count；WBC, white blood cell; PLT, platelet count; NEU, neutrophil count; EOS, eosinophils；LYM, lymphocyte count; MONO, monocyte count; RBC, red blood cell；Hb，hemoglobin；Fib，fibrinogen；TP，total protein ；ALB，albumin；INR, international normalized ratio; ALT, alanine transaminase; AST, aspartate transaminase; TBILtotal bilirubin；IBIL，indirect bilirub；CR, Creatinine; BUN, Blood urea nitrogen; UA, uric acid; GLU, glucose; Na, sodium ; K, potassium; Cl, chloride; Mg, magnesium; Ca, calcium; TC, total cholesterol; TG, triglycerides; HDL, high density lipoprotein; LDL, low density lipoprotein; ApoA, apolipoprotein A; ApoB，apolipoprotein B；hosp_day, length of hospital stay for enrolled patients; Values with *P* <0.05 are in bold.
